# Ciprofloxacin, amoxicillin, and aminoglycosides stimulate genetic and phenotypic changes in uropathogenic *Escherichia coli* strains

**DOI:** 10.1080/21505594.2019.1596507

**Published:** 2019-04-02

**Authors:** Wioletta Adamus-Białek, Monika Wawszczak, Michał Arabski, Michał Majchrzak, Martyna Gulba, Dariusz Jarych, Paweł Parniewski, Stanisław Głuszek

**Affiliations:** a Department of Surgery and Surgical Nursery with Laboratory of Genetics, Faculty of Medicine and Health Sciences, Jan Kochanowski University, Kielce, Poland; b Department of Biochemistry & Genetics, Jan Kochanowski University, Kielce, Poland; c Institute of Medical Biology, Polish Academy of Sciences, Łódź, Poland

**Keywords:** Uropathogenic *Escherichia coli* Strains (UPEC), antibiotics, virulence factor genes, biofilm

## Abstract

Antibiotic therapy and its consequences in bacterial and human aspects are widely investigated. Despite this, the emergence of new multidrug resistant bacteria is still a current problem. The scope of our work included the observation of changes among uropathogenic *Escherichia coli* strains after the treatment with a subinhibitory concentration of different antibiotics. The sensitive strains with or without virulence factors were incubated with amoxicillin, ciprofloxacin, gentamycin, or tobramycin. After each passage, the *E. coli* derivatives were compared to their wild types based on their susceptibility profiles, virulence genes, biofilm formations and the fingerprint profiles of PCR products amplified with using the (N)(6)(CGG)(4) primer. It turned out that antibiotics caused significant changes in the repertoire of bacterial virulence and biofilm formation, corresponding to acquired cross-resistance. The genomic changes among the studied bacteria were reflected in the changed profiles of the CGG-PCR products. In conclusion, the inappropriate application of antibiotics may cause a rapid rise of Multidrug Resistant (MDR) strains and give bacteria a chance to modulate their own pathogenicity. This phenomenon has been easily observed among uropathogenic *E. coli* strains and it is one of the main reasons for recurrent infections of the urinary tract.

## Introduction

Extensive research provides increasingly strong evidence of the importance of uropathogenic *Escherichia coli* (UPEC) strains for medical and epidemiological problems. Their remarkable adaptive abilities are conductive to acute or chronic urinary tract infections (UTI) and cause serious therapeutic problems [1,2]. Nowadays they are not only seen as *E. coli* isolated from urine, but a specialized pathotype with specific mechanisms of intracellular pathogenicity, which allow for colonization of the urinary tract, avoidance of host defenses and causing damage to the uroepithelium [3–6]. In addition, a high spread of antibiotic resistance is observed within these pathotypes [7–12]. They owe this high capacity to genetic modulate, own pathogenicity and drug resistance [13–15]. Several urovirulence factors, such as hemolysin (the *hly* gene), the cytotoxic necrotizing factor type 1 (the *cnf-1* gene), the P-pili (the *pap* genes), S-family adhesions (the *sfa* gene), and the bacteriocin usp (the *usp* gene) are coded by the genes located on pathogenicity islands (PAIs) and through this they can be quickly transferred via the horizontal gene transfer [16–20]. This mechanism is also observed during the transfer of the genes conditioning resistance to some antibiotics. Numerous antibiotics are recommended for the treatment of UTI [21–23]. They belong mainly to betalactams and cephalosporins, but aminoglycosides and fluoroquinolones are also often applied [24]. In response to this – UPEC strains developed a lot of defense mechanisms against antibiotics, related to changes in protein expression or gene mutations. Furthermore, the loss of certain parts of UPEC PAIs was observed during the incubation with ciprofloxacin. This phenomenon is related to the induction of the SOS response (to global DNA damage in bacteria) induced by some antibiotics [25,26]. Epidemiological studies revealed that the strains with a higher drug-resistance had a lower number of virulence genes in contrast to the strains with lower drug-resistance [27]. It is an established fact that the mechanisms of drug resistance belong to complex mechanisms of bacterial adaptation, regulated by many factors [2,28–30]. The situation becomes more complicated when the antibiotic is incorrectly applied, especially during ambulatory therapy. The problem is that when the antibiotic reaches a subinhibitory concentration before the delivery of the next dose, it gives bacteria an opportunity to develop a drug resistance and modulate their own pathogenicity [31]. We would like to consider these hypotheses *in vitro*, including different antibiotics and properties of the UPEC strains.

## Materials and methods

### Bacterial strains

Five clinical *Escherichia coli* strains isolated from the urine of patients suffering from urinary tract infection were selected for this study. They belong to the characterized bacterial collection used in previous studies [30,32,33]. The basic criteria for their selection were the sensitivity to all analyzed antibiotics and the varied occurrence of virulence factor genes. The characteristic of these strains was presented in Table 1. *E. coli* ATCC 25,922 was used as the control strains during antimicrobial susceptibility testing according to the guidelines of the European Committee on Antimicrobial Susceptibility Testing (EUCAST).

**Table 1.** The characteristic of clinical uropathogenic *E. coli* strains selected for the study; 0 – lack of the gene, 1 – gene presence, S – sensitive, I – intermediately sensitive. CIP – ciprofloxacin, NOR – norfloxacin, OFX – ofloxacin, AML – amoxicillin, AMC – amoxicillin/clavulanate, PRL – piperacillin, CAZ – ceftazidime, FOX – cefoxitin, CTX – cefotaxime, IMI – imipenem, GN – gentamycin, TN – tobramycin, AK – amikacin, NET – netilmicin, NI – nitrofurantoin, W – trimethoprim, SXT – trimethoprim/sulfamethoxazole.

*E. coli* wild-type strains		1	5	6	16	84
Phylogenetic group		A	A	B	B	B
Biofilm formation level (A_531_)		0.09	0.07	0.1	0.09	0.06
Virulence factors’ genes	*papC*	0	0	0	**1**	**1**
	*sfaE/D*	0	0	**1**	**1**	**1**
	*cnf1*	0	0	**1**	0	**1**
	*usp*	0	0	**1**	**1**	**1**
	*fimG/H*	**1**	**1**	**1**	**1**	**1**
	*hlyA*	0	0	**1**	0	**1**
Drug-resistance genes	*bla_TEM-1_*	0	0	0	0	0
	*sul1*	0	1	0	0	0
	*sul2*	0	1	0	0	0
	*bla_CTX-M1_*	0	0	0	0	0
	*bla_SHV_*	0	0	0	0	0
	*bla_OXA-1_*	0	0	0	0	0
	*bla_CMY_*	0	0	0	0	0
	*aac-(3)-II*	0	0	0	0	0
**Drug-**resistance profiles	CIP	S	S	S	S	S
	NOR	S	S	S	S	S
	OFX	S	S	S	S	S
	AML	S	S	S	S	S
	AMC	S	S	S	S	S
	PRL	S	S	S	S	S
	CAZ	S	I	S	S	S
	FOX	S	S	S	S	S
	CTX	S	S	S	S	S
	IMI	S	S	S	S	S
	GN	S	I	I	I	S
	TN	S	I	I	I	S
	AK	S	S	S	S	S
	NET	I	S	I	I	S
	NI	S	S	S	S	S
	W	S	S	S	S	S
	SXT	S	S	S	S	S

### Experiment plan

**Figure 1.**
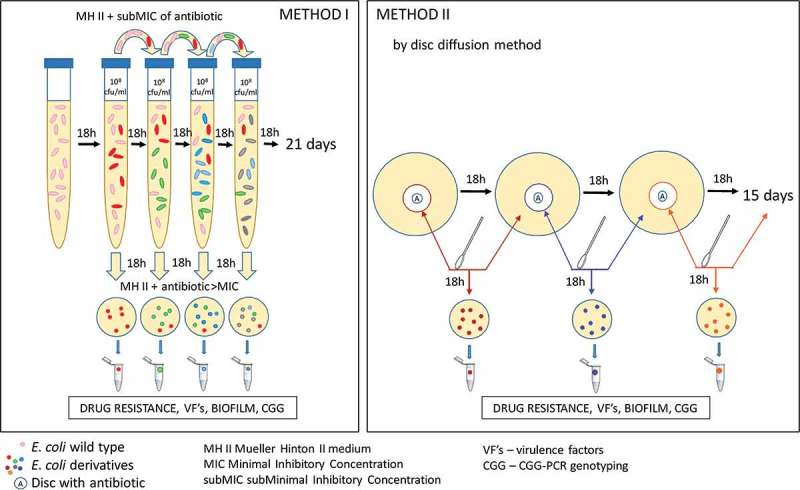
The schematic description of the *E. coli* derivatives selection using Method I and Method II described in the Materials and Methods.

The process of selection of derivatives from *E. coli* wild-type strains consisted of different stages and it was dependent on the antibiotics used. In case of ciprofloxacin and amoxicillin, the experiment was performed in liquid broth (Method I). Because of the insolubility of tobramycin and gentamicin in broth – the derivatives were selected using the agar (Method II). The schematic process of the experiments was presented in Figure 1. After the selection of *E. coli* derivatives, their antibiotic susceptibility profiles were analyzed via the Disc Diffusion Method. Resistant derivatives of *E. coli* were further analyzed. Their growth curves under the antibiotic pressure were compared to the standard logarithmic growth of the control strains (*E. coli* ATCC 25,922). Next, the stability of the generated drug resistance among derivatives was evaluated as well. Additionally, the selected derivatives of *E. coli* were verified using the E.coli Chromogenic Medium (Biocorp). Further, derivatives were analyzed based on the presence and expression of virulence factors genes and biofilm formation. Finally, the CGG-PCR profiles between derivatives and their wild type strains were compared.

### *E. coli* derivatives selection – Method I

The minimal inhibitory concentrations (MICs) of antibiotics (ciprofloxacin and amoxicillin) were determined for each *E. coli* strain in accordance with the serial dilution method. Bacterial suspensions in 0.9% NaCl were prepared from overnight cultures. The turbidity was equal to 0.5 in the McFarland standard (∼1 × 10^8^ CFU/ml). The prepared suspensions were diluted to ∼1 × 10^6^ CFU/ml in Mueller Hinton II (MHII) broth with varying concentrations of the antibiotic to the final volume of 4 ml and they incubated for 18 h in 37°C. The purity of broth was verified via swabbing of 100 µl of sterile broth onto MHII Agar plates. According to the definition of the MIC, we designated MIC as the starting point and the boundary for the determination of subminimal inhibitory concentrations (sub-MIC) of particular antibiotics. We assumed the sub-MIC to be the highest concentrations of the antibiotic in which the bacterial growth is observed. Optimized sub-MICs were further used in these studies and they were respectively: 0.008 mg/L of ciprofloxacin for *E. coli* No. 5, No. 16, and No.84; 0,016 mg/L of ciprofloxacin for *E. coli* No. 1 and No. 6; 4 mg/L of amoxicillin for *E. coli* No. 1, No. 5, No. 6, and No. 84; 8 mg/L of amoxicillin for *E. coli* No. 16.

The 40 µl of overnight bacterial inoculum was transferred to the MH II broth supplemented sub-MIC of antibiotic in a total volume of 4 ml, to the final culture concentration of ∼1 × 10^6^ CFU/ml. After 18 ± 2 h of incubation, the 40 µl of bacterial suspension was passed on to the next broth with the sub-MIC of the antibiotic. The passes were conducted according to this scheme for 21 days. *E. coli* wild-type strains were passed on the same way without the antibiotic and they were considered as negative control for the experiment. Simultaneously, each overnight culture was swabbed onto a MH II Agar supplemented with the antibiotic >MIC (amoxicillin >10 mg/L, ciprofloxacin >1.5 mg/L according to EUCAST MIC tables, 2016), toward the selection of antibiotic-induced derivatives. The grown colony was randomly selected as the *E. coli* derivative and it was multiplied and prepared in glycerol stocks to be stored in −80°C. The following analyses were conducted for the characterization of the selected derivatives.

### *E. coli* derivatives selection – Method II

*E. coli* derivatives were selected using the MH II Agar with the presence of standard antibiotic disks (10µg of Gentamycin, 10 µg of Tobramycin). The experiment was prepared according to the standard disc diffusion method (EUCAST, 2018). After each overnight (18 h) bacterial incubation, the 0.5 McFarland bacterial suspension in 0.9% NaCl was prepared through the collection of bacteria from the border of the growth inhibition zone around the antibiotic disc. Simultaneously, the same swab was used for the semiquantitative bacterial culture incubated under the same conditions on the MH II agar. During each passage, the zone of growth inhibition was measured and compared to the EUCAST standards. After exceeding the limit value, the culture was classified as resistant and the *E. coli* derivative was selected by random isolation of a single grow colony on the second plate of the MH II agar. The passing of *E.coli* wild type strains without antibiotic discs was considered as a negative control for the experiment. All passes were conducted for 15 days and the isolated single colonies of *E. coli* derivative were multiplied and prepared in glycerol stocks to be stored in −80°C. The following analyses were conducted for the characterization of the selected derivatives.

### Antimicrobial susceptibility testing

Antimicrobial susceptibility testing was carried out via the disk diffusion method on Mueller–Hinton II Agar plates, using commercial disks (Oxoid, Wesel, Germany) according the protocol described previously [33]. The *E. coli* derivatives and wild-type strains were tested against 17 antimicrobiotics: amoxicillin (AML, 25 μg), amoxicillin/clavulanate (AMC, 20 + 10 μg), piperacillin (PRL, 30 μg), cefoxitin (FOX, 30 μg), cefotaxime (CTX, 5 μg), ceftazidime (CAZ, 10 μg), imipenem (IMP, 10 μg), amikacin (AK, 30 μg), tobramycin (TN, 10 μg), gentamicin (GN, 10 μg), netilmicin (NET, 10 μg), norfloxacin (NOR, 10 μg), ciprofloxacin (CIP, 5 μg), ofloxacin (OFX, 5 μg), trimethoprim (W, 5 μg), trimethoprim-sulfamethoxazole (STX, 25 μg), and nitrofurantoin (NI, 100 μg). The susceptibility testing of bacteria strains was interpreted according to the EUCAST guidelines based on the values of the growth inhibition zones. Bacterial strains were determined as sensitive (S), intermediately sensitive (I), or resistant (R) to the particular antibiotics. The analysis was repeated three times for 10 randomly selected strains.

### Growth curves

A fresh inoculum of bacteria was prepared in the MH II Broth at 37°C for 24 h. The bacterial culture was diluted to obtain 0.125 ± 0.005 OD at 600 nm using the Microplate Reader (TECAN Infinite 200 PRO, Tecan Group Ltd., Switzerland), corresponding to approximately 10^3^ CFU of bacteria. The *E. coli* derivatives were grown in triplicate on 96-well plates at 37°C in the LB broth with a sub-MIC of the antibiotic. The negative control was the wild-type *E. coli* strains incubated under the same conditions. The positive control for standard logarithmic growth curves was *E. coli* ATCC 25,922 cultured in the optimal conditions. The bacterial growth was measured at 600 nm using a Microplate Reader after every 2 h of incubation for 24 h. All measurements were performed in two independent experiments.

### Resistance stability assay

*E. coli* derivatives resistant to ciprofloxacin, amoxicillin, or tobramycin were passaged on to the MH II Agar broth in optimal conditions (without antibiotics) for 19 days (derivatives generated by ciprofloxacin and amoxicillin) and 8 days (derivatives generated by tobramycin). After the 1^st^, 4^th^, 8^th^, 12^th^, and 19^th^ day of passage, the bacterial susceptibility profiles for 17 antimicrobials were determined.

### PCR and gene expression

The genomic DNA isolated from the fresh inoculum of the bacteria was used for the identification of the studied genes (*papC, sfaD/sfaE, cnf1, usp, fimG/H*, and *hlyA*). The specific PCR parameters for all primers used in the study and their references are shown in Table 2. PCRs were performed with bacterial DNA (approx. 20 ng) in a 25 µl reaction mixture containing a 12.5 µl DreamTaq™ Green DNA Polymerase Master Mix (2×) (ThermoFisher Scientific™) and 10 pmol of each primer (oligo.pl) and refilled with MiliQ water. An individual adjustment of the conditions of DNA amplification was carried out in am Eppendorf thermocycler. After electrophoresis on 2% agarose gel, the PCR products were visualized under UV.

**Table 2.** Oligonucleotides used in the study.

Primer	Sequence (5ʹ → 3ʹ)	Locus	Ta [°C]	PCR [bp]	Ref.
Pap1 Pap2	GACGGCTGTACTGCAGGGTGTGGCG ATATCCTTTCTGCAGGGATGCAATA	*papC*	60	328	Adamus-Bialek et al., 2009
Sfa1 Sfa2	CTCCGGAGAACTGGGTGCATCTTAC CGGAGGAGTAATTACAAACCTGGCA	*sfaD/sfaE*	60	410	
Cnf1a Cnf2a	AAGATGGAGTTTCCTATGCAGGAG CATTCAGAGTCCTGCCCTCATTATT	*cnf1*	60	498	
Usp1mod Uspe2mod	TTCTGGGGAACTGACATTCACGG CCTCAGGGACATAGGGGGAA	*usp*	60	657	
FimGH1 FimGH2	GCAATGTTGGCGTTCGCAAGTGC CGTAAATATTCCACACAAACTGG	*fimG/H*	60	1001	
Hly1mod Hly2mod	AACAACGATAAGCACTGTTCTGGCT ACCATATAAGCGGTCATTCCCATCA	*hlyA*	60	1177	

Qualitative gene expression was evaluated at the transcription level. The total mRNA was isolated by the FastRNA^TM^ Spin Kit (MP Biomedica) using the FastPrep® Instrument (MP Biomedica). Next, the cDNA was synthesized using the TranScriba Kit (A&A Biotechnology) and the presence of particular genes was detected with the PCR standard method described above.

### Biofilm formation assay

The biofilm formation assay was carried out by 0.3% crystal violet staining according to the protocol described previously [32]. The bacteria were grown in triplicate on the 96-well plates at 37°C in the LB broth. A medium without bacteria incubated under the same conditions was used as a negative control. Biofilm formation was measured at 531 nm using a Microplate Reader. All measurements were performed in two independent experiments. A blank corrected mean absorbance value of <0.04 from the negative control was considered as a biofilm-negative strain. The results were normalized to verify if the biofilm formation is independent from the bacterial growth level. B_Rel_ (Biofilm relative) was estimated based on the ratio between the absorbance level of the formed biofilm and the absorbance level of the growth (A_531_/A_600_). Additionally, the biofilm formation stability was measured for derivative strains with increased biofilm formation. The bacterial cultures at control conditions (24 h, 37°C in LB broth without antibiotics) were passaged on for 20 days in triplicate on the 96-well plates. After each day of passing, the fresh bacterial inoculum was measured for biofilm formation according to the protocol described above.

### (CGG)_4_-based PCR

The DNA fingerprint profiles of particular *E. coli* strains were obtained according to the protocol described in the previous study [30] except for the use of a different polymerase and 4 min of elongation. The products of PCR were amplified by using 100 pmol of N_6(_CGG)_4_ added to the reaction mixture containing bacterial DNA (approx. 20 ng) and the 12.5 µl DreamTaq™ Green DNA Polymerase Master Mix (2x) (ThermoFisher Scientific™) and filled with MiliQ water up to 25 µl of total volume. The 1^st^ stage of PCR was denaturation at 95°C for 3 min, then repeated 35 times at 95°C for 1 min and at 72°C for 4 min, followed by a final extension step at 72°C for 8 min. After the electrophoresis on 3% agarose gel, the CGG-PCR products were visualized under UV.

### Statistical analyses

The differences in antibiotic susceptibility between wild types and derivatives of studied *E. coli* strains were analyzed statistically using a two-tailed, paired *T*-test, where *p* < 0.05 meant statistically significant. The statistically significant (*p* < 0.05) difference between the biofilm formation level was calculated based on a two-tailed, unpaired *T*-test. GraphPad Prism, version 6 (San Diego, CA, USA) was used for the analyses and derivation of figures.

## Results

### Antibiotic sensitivity of *E. coli* derivatives

Five uropathogenic clinical *E. coli* strains (Table 1) were chosen for the experiments. In total, the 121 *E. coli* derivative strains were generated after the treatment with a subinhibitory concentration of different antibiotics. Particular groups of derivatives exhibited the ability to grow above the MIC of ciprofloxacin, amoxicillin, gentamicin or tobramycin. In the case of the selected derivatives that did not confirm the resistance to the antibiotic via the disc diffusion method – they were identified as antibiotic-tolerant derivative strains. The characterization of the general antibiotic impact on the rate of changes among the studied bacteria was presented in Table 3. During the selection process – the day selection of the first derivative was compared to the day of selection of the first derivative with confirmed antibiotic resistance. Also, the total number of *E. coli* derivatives induced by particular antibiotics was presented. Ciprofloxacin induced the resistance the fastest, all strains (except *E. coli* No. 84) generated four resistant derivatives just after the first day of passage. *E. coli* No. 84 did not generate any derivatives upon treatment with ciprofloxacin. Another antibiotic that quickly induced the resistance was amoxicillin – just after the second day of passage, the colonies appeared on the agar with the antibiotic above the MIC, except *E. coli* No. 84 which generated the first antibiotic-tolerant derivative strain after the sixth day of passage. In case of aminoglycosides, the first derivatives appeared between the third and the fifth day of passage. Usually, except for induction by ciprofloxacin, the resistance appeared successively later than tolerance to antibiotics. The largest number of derivatives was obtained in the culture with the sub-MIC of amoxicillin (42), the lowest number of *E. coli* derivative strains was generated upon treatment with gentamycin, but the differences between this antibiotic and tobramycin and ciprofloxacin were slight. None of the wild-type *E. coli* strains acquired the antibiotic resistance during the control passes (cultures without antibiotic).

**Table 3.** The rate of derivatives and drug-resistance generation. The day of the 1^st^ derivative appearance was confirmed according to developed method I for CIP and AML and method II for GN and TN, described in the Materials and Methods. The day of the first resistance of particular derivatives was confirmed via the disc diffusion method. The total number of all selected derivatives represents the strains expressing antibiotic-tolerance >MIC of ciprofloxacin (CIP), amoxicillin (AML), gentamycin (GN), or tobramycin (TN).

	Day of first derivative/Day of first resistance/Total number of selected derivatives generated by:			
*E. coli* wild type	CIP	AML	GN	TN
1	**1**/**1**/**7**	**2**/**2**/**9**	**4**/**8**/**5**	**4**/**6**/**6**
5	**1**/**1**/**7**	**2**/**2**/**9**	**3**/**0**/**5**	**3**/**5**/**6**
6	**1**/**1**/**7**	**2**/**5**/**8**	**5**/**0**/**4**	**4**/**6**/**6**
16	**1**/**1**/**7**	**2**/**13**/**8**	**3**/**7**/**5**	**3**/**7**/**6**
84	**0/0/0**	**6**/**0**/**8**	**3**/**0**/**3**	**6**/**0**/**5**

**Figure 2.**
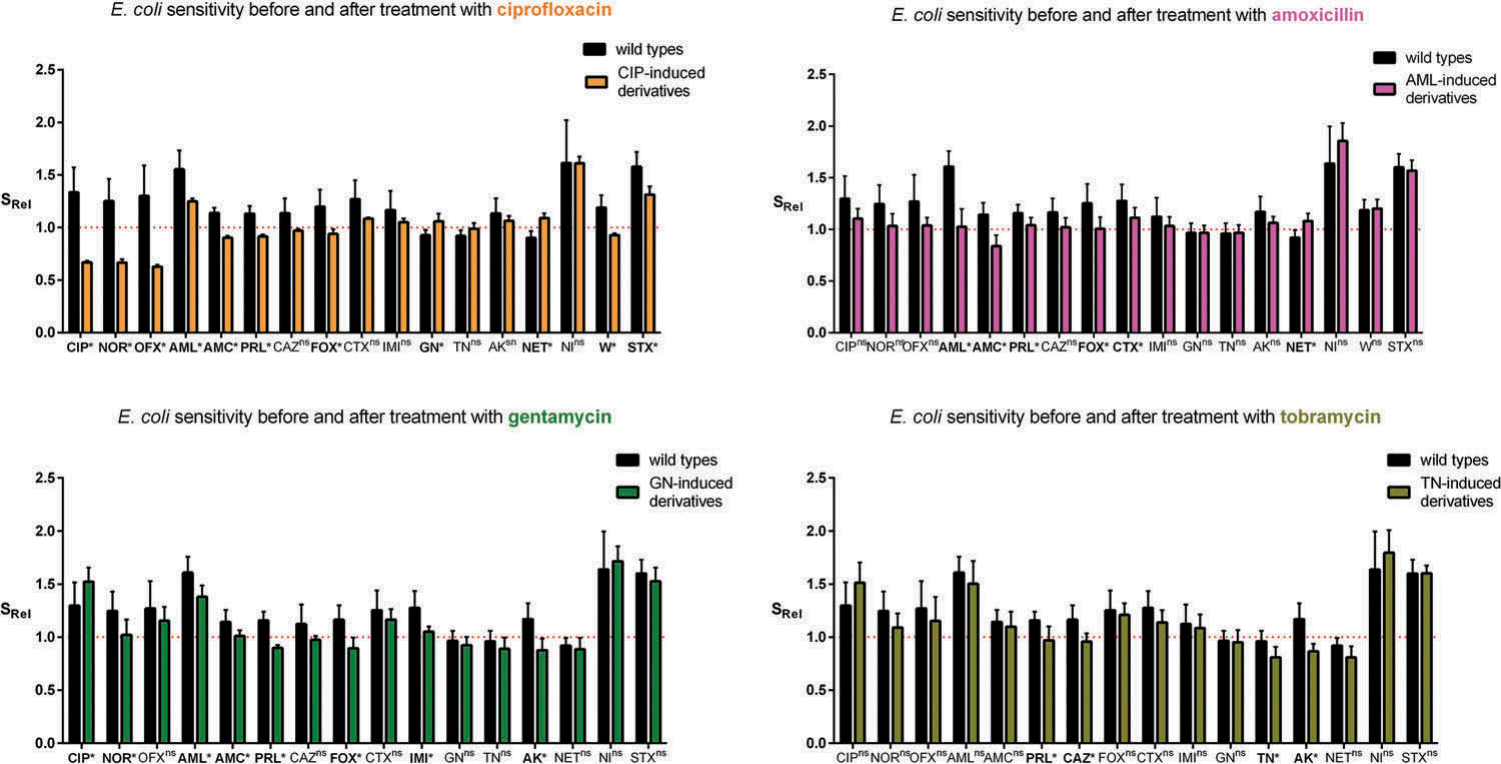
The relative antibiotic sensitivity (S_Rel_) of wild-type *E. coli* strains (No. 1, No. 5, No. 6, No. 16, and No. 84) and their derivatives isolated during the treatment with a subinhibitory concentration of antibiotics (ciprofloxacin, amoxicillin, gentamycin, and tobramycin). The sensitivity was measured for all studied antibiotics (CIP – ciprofloxacin, NOR – norfloxacin, OFX – ofloxacin, AML – amoxicillin, AMC – amoxicillin/clavulanate, PRL – piperacillin, CAZ – ceftazidime, FOX – cefoxitin, CTX – cefotaxime, IMI – imipenem, GN – gentamycin, TN – tobramycin, AK – amikacin, NET – netilmicin, NI – nitrofurantoin, W – trimethoprim, SXT – trimethoprim/sulfamethoxazole). The results (S_Rel_) represent the ratio between the medium value of diameter zone of inhibited growth of *E. coli* strains (wild type or derivatives) and the limit value of the sensitivity diameter zone according to the clinical breakpoint tables from EUCAST 2018. The value 1 on the Y axis means the limit value of the diameter zone of inhibited bacterial growth corresponding to sensitivity according to the clinical breakpoint tables of EUCAST 2018. The statistically significant (*p* < 0.05)* difference between the sensitivity to individual antibiotics was determined using a two-tailed, paired *T*-test (GraphPad Prism, version 6; San Diego, CA, USA), ns – not statistically significant.

Subsequently, all selected strains grouped into ciprofloxacin-induced derivatives, amoxicillin-induced derivatives, gentamycin-induced derivatives, and tobramycin-induced derivatives were compared to their wild types of *E. coli* strains based on the antibiotic sensitivity (Figure 2). To equalize the sensitivity to antibiotics in relation to the border of resistance, the relative values of sensitivity (S_Rel_) were estimated. The sensitivity of the derivatives to almost all antibiotics (excluding mainly amoxicillin, nitrofurantoin, trimethoprim-sulfamethoxazole) was decreased and it was close to the border of sensitivity. The level of sensitivity variation to antibiotics was observable in particular groups of derivative *E. coli* strains. The statistically significant changes of antibiotic sensitivity among derivatives were observed mostly for ciprofloxacin-induced derivatives. In the case of 11 out of 17 antibiotics, the bacterial sensitivity was significantly changed, the sensitivity mostly decreased, except gentamicin and netilmicin where the sensitivity increased. A similar correlation was observed in the case of gentamicin-induced derivatives, which exhibited an increased sensitivity to ciprofloxacin. Increased sensitivity was observed among amoxicillin-induced derivatives also regarding netilmicin. The strongest decrease of sensitivity was observed toward fluoroquinolones among ciprofloxacin-induced derivatives. The resistance to amoxicillin (amoxicillin-induced derivatives) was correlated with significantly decreased sensitivity only to betalactam antibiotics (amoxicillin/clavulanate, piperacillin, cefoxitin, and cefotaxime). The aminoglycosides used for the selection of derivatives caused similar changes in the sensitivity to the analyzed antibiotics. However, gentamycin significantly decreased the sensitivity to six betalactam antibiotics and norfloxacin, whereas tobramycin significantly decreased only the sensitivity to piperacillin. To sum up, it was observed that the antibiotics used for the selection of *E. coli* derivatives induced resistance to other antibiotics. However, the increased sensitivity to antibiotics with correlation to increased resistance to other antibiotics was also observed in some cases. The cross-resistance was especially observed in the case of ciprofloxacin (Table 4). Ciprofloxacin induced the resistance to all fluoroquinolones, amoxicillin/clavulanate and trimethoprim among 100% of derivatives and induced 64% of cefoxitin-resistant derivatives. The other antibiotics (AML, GEN, and TOB) induced the cross-resistance to more antibiotics, but in a smaller number of derivative strains. The resistance to fluoroquinolones did not correspond with aminoglycoside resistance, as it was observed in Figure 2. Furthermore, the sensitivity to aminoglycosides was correlated with the resistance to ciprofloxacin and vice versa. Nonetheless, the aminoglycosides have induced resistance mainly to aminoglycosides, also but to a lesser extent to betalactams and to fluoroquinolones. All derivative strains remained sensitive to imipenem and also in majority they remained sensitive to cefotaxime, trimethoprim/sulfamethoxazole and nitrofurantoin. To sum up, ciprofloxacin induced the resistance to at least one antibiotic from 17 analyzed among 39% of derivatives, while other antibiotics (amoxicillin, gentamycin, or tobramycin) induced the resistance to other antibiotics only in a dozen or so % of derivatives. On the other hand, ciprofloxacin never induced the resistance in 10 out of 17 antibiotics, aminoglycosides – 6–7 and amoxicillin – 5.

**Table 4.** Heat map of the percentage of *E. coli* derivatives with acquired resistance to particular antibiotics. I – Fluoroquinolones, II – Penicillins, III – Cephalosporins, IV – Carbapenems, V – Aminoglycosides, VI – Others, CIP – ciprofloxacin, NOR – norfloxacin, OFX – ofloxacin, AML – amoxicillin, AMC – amoxicillin/clavulanate, PRL – piperacillin, CAZ – ceftazidime, FOX – cefoxitin, CTX – cefotaxime, IMI – imipenem, GN – gentamycin, TN – tobramycin, AK – amikacin, NET – netilmicin, NI – nitrofurantoin, W – trimethoprim, SXT – trimethoprim/sulfamethoxazole, NT – not tested, Avg – average of percentage of *E. coli* derivatives with acquired resistance to all antibiotics.

**Figure 3.**
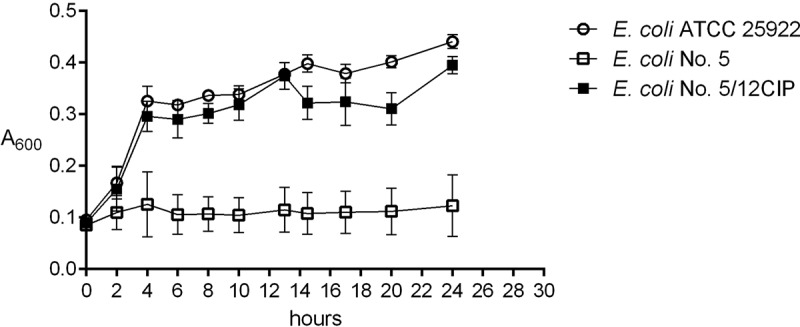
The examples of growth curves of the wild type (*E. coli* No. 5, negative control) and its derivative (*E. coli* No. 5/12CIP) upon treatment with the sub-MIC of antibiotics (amoxicillin or ciprofloxacin). The positive control of logarithmic growth curve was *E. coli* ATCC 25,922 incubated in optimal conditions. The absorbance level of the growth (A_600_) was measured every two hours. Each bar represents the mean with standard deviation, all measurements were performed in two independent experiments.

Additionally, the growth curves of randomly selected 9 *E. coli* derivatives (No.: 1/20CIP, 5/12CIP, 6/4CIP, 16/20CIP, 1/10AML, 5/2AML, 6/15AML, 16/11AML, and 84/21AML) were examined during the incubation with the sub-MIC of amoxicillin or ciprofloxacin. The derivative strains started growth at the same time as the control *E. coli* ATCC 25,922 and their growth curves remained at the same level. The difference was very slight. The wild type of *E. coli* strains was unable to grow under the treatment with the sub-MIC of antibiotics. The example of the growth curve of the derivative strain in comparison to the negative and positive control were presented in Figure 3. Next, the stability of induced antibiotic resistance was verified for 10 antibiotic-resistant derivatives generated by ciprofloxacin, amoxicillin, and tobramycin (Table 5). Generally, the most stable resistance was discovered in the case of derivative strains generated by ciprofloxacin. All derivatives maintained resistance to all tested quinolones, even after 19 days of incubation. It was also noted that 85% of these derivative strains remained resistant to amoxicillin/clavulanate. Variably stable resistance was observed among the group of amoxicillin-resistant derivatives. Interestingly, during the incubation without the sub-MIC of amoxicillin, all the tested derivatives lost their resistance to amoxicillin just after the first day of passage, but they remained resistant to amoxicillin/clavulanate. The least stable resistance was observed in the group of tobramycin-induced derivatives. The resistance to tobramycin remained among all the tested derivatives for all days of passage, however a complete loss of resistance was observed in case of the other aminoglycosides.

**Table 5.** The stability of acquired resistance among derivatives of *E. coli* strains. After the 1^st^, 4^th^, 8^th^, 12^th^, and 19^th^ day of passage of derivatives the antibiotic resistance was verified by disc diffusion method. High stability – 100% of derivatives remained resistant; *85% of derivatives remained resistant; diverse stability – the resistance profiles of the derivatives were diverse after the resistance stability assay; instability – 100% of derivatives lost the drug resistance.

E. coli derivatives selected by:	Induced antibiotic resistance to:	Drug-resistance stability
Ciprofloxacin	Quinolones	High stability
	Amoxicillin/Clavulanate	High stability*
	Cefoxitin	Diverse stability
	Trimethoprim	Diverse stability
Amoxicillin	Amoxicillin,	Instability
	Amoxicillin/Clavulanate	High stability
	Cefoxitin	Diverse stability
Tobramycin	Tobramycin	High stability
	Gentamicin	Instability
	Amikacin	Instability
	Netilmicin	Instability

### Gene loss

The analysis of gene loss such as *papC, sfaE/D, cnf1, usp, fimG/H*, and *hlyA* was examined in all derivatives (67) adequately to their wild type *E. coli* strains (No. 6, No. 16, and No. 84). Only ciprofloxacin-induced derivatives (13; 19%) demonstrated the loss of particular genes, other derivative strains kept their original genes profiles (Table 6). The *E. coli* No. 84 have not acquired the resistance to ciprofloxacin, so none CIP-derivative of No. 84 was generated. Ciprofloxacin-induced derivatives kept or lost the virulence factor genes variously, however all derivatives kept the *fimG/H*. Only one derivative (*E. coli* 6/7CIP) kept their original profile of virulence genes. The remaining derivatives of *E. coli* No. 6 have lost all the other analyzed genes. In the case of the derivatives of *E. coli* No. 16, the virulence gene profiles were differential. All derivative strains have lost *papC*, 5 out of 7 derivative strains have lost *usp* and 3 out of 7 have lost *sfaE/D*.

**Table 6.** The occurrence of virulence factor genes (*papC, sfaD/E, cnf1, usp, fimG/H*, and *hlyA*) in ciprofloxacin-induced *E. coli* derivatives and their wild type *E. coli* strains; 0 – lack of the gene, 1 – gene presence. The lack or presence of particular genes was consistent with no or active expression on the transcriptional level.

*E. coli* strains		Genes					
Wild type	Derivative	*papC*	*sfaD/E*	*cnf1*	*usp*	*fimG/H*	*HlyA*
6		0	1	1	1	1	1
	6/1CIP	0	0	0	0	1	0
	6/4CIP	0	0	0	0	1	0
	6/7CIP	0	1	1	1	1	1
	6/10CIP	0	0	0	0	1	0
	6/12CIP	0	0	0	0	1	0
	6/16CIP	0	0	0	0	1	0
	6/20CIP	0	0	0	0	1	0
16		1	1	0	1	1	0
	16/1CIP	0	1	0	1	1	0
	16/4CIP	0	1	0	0	1	0
	16/7CIP	0	0	0	0	1	0
	16/10CIP	0	1	0	1	1	0
	16/12CIP	0	0	0	0	1	0
	16/16CIP	0	0	0	0	1	0
	16/20CIP	0	1	0	0	1	0

**Figure 4.**
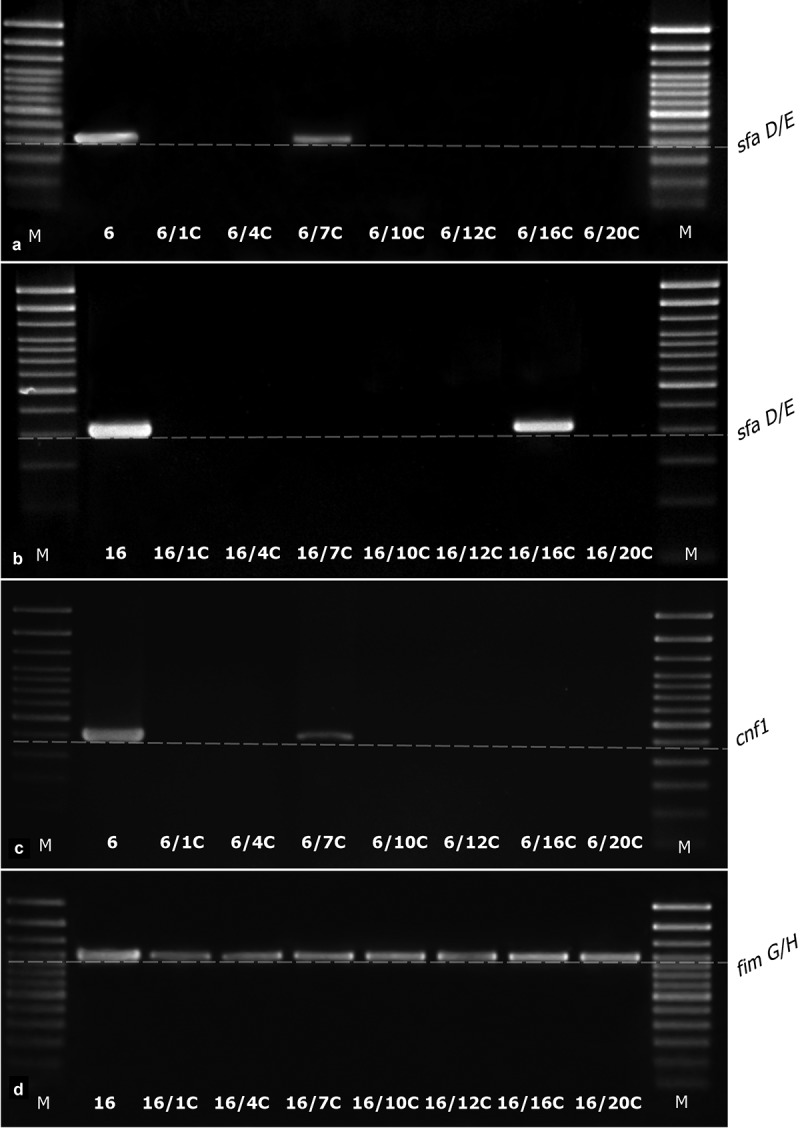
The examples of agarose gel electrophoresis for PCR products of *sfaD/E* (A, B), *cnf1* (C), and *fimG/H* (D) gene fragments amplified from cDNA synthesized by reverse transcription of mRNA isolated from *E. coli* wild type (No. 6 and No. 16) and ciprofloxacin-resistant derivatives (No.: 6/1C, 6/4C, 6/7C, 6/10C, 6/12C, 6/16C, 6/20C, 16/1C, 16/4C, 16/7C, 16/10C, 16/12C, 16/16C, and 16/20C), M – 100 bp DNA Ladder (Invitrogen).

The observed loss of genes corresponded with the loss of their expression on the transcription level (Figure 4). None of the bacterial colonies of wild-type strains (randomly selected) isolated during passages in control conditions have lost the analyzed genes.

### Biofilm formation

**Figure 5.**
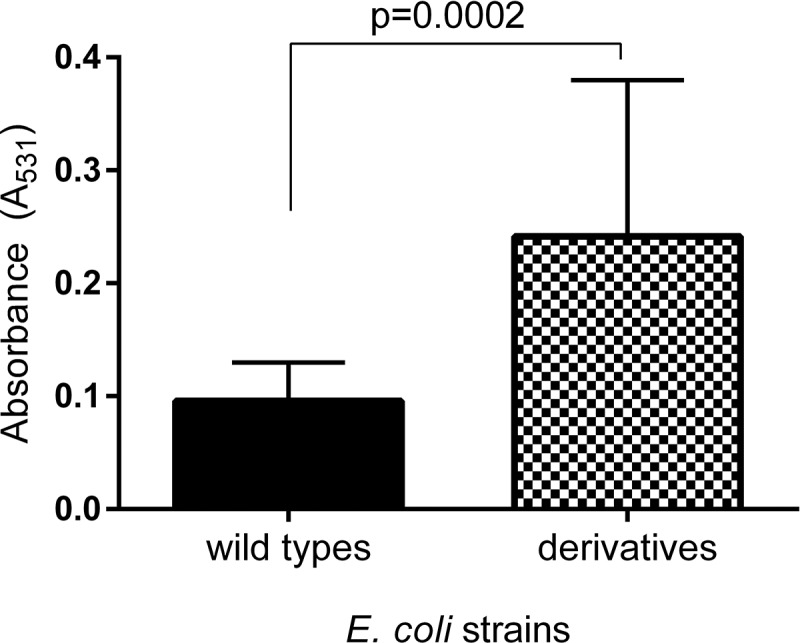
The comparison of the biofilm formation between the wild types and derivatives (amoxicillin-induced) of *E. coli* strains No. 5 and No. 6. Each bar represents the mean with standard deviation based on the OD value of the absorbed crystal violet (0,3%) measured at 531 nm (A_531_) for each strain from particular groups (wild types and derivatives). The study was performed in four replications in two independent experiments. The difference is significant (*p* < 0.05, unpaired *T*-test, two-tailed, nonparametric).

**Figure 6.**
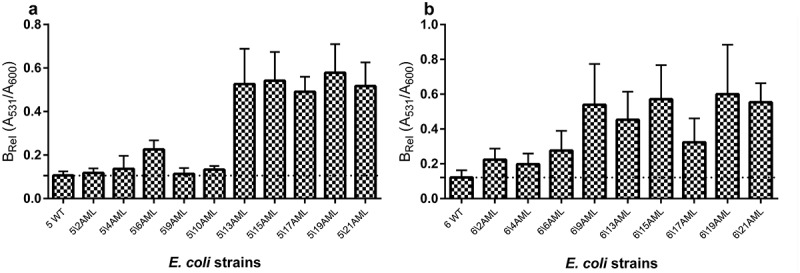
The relative biofilm formation of the wild type and derivatives (amoxicillin-induced) of *E. coli* strain No 5. (A) and No. 6 (B). The biofilm was analyzed based on the OD value of the absorbed crystal violet (0,3%) measured at 531 nm (A_531_). The results represent the values of relative biofilm formation (B_Rel_) independent of bacterial growth (A_531_/A_600_). The study was performed in four replications, in two independent experiments.

**Figure 7.**
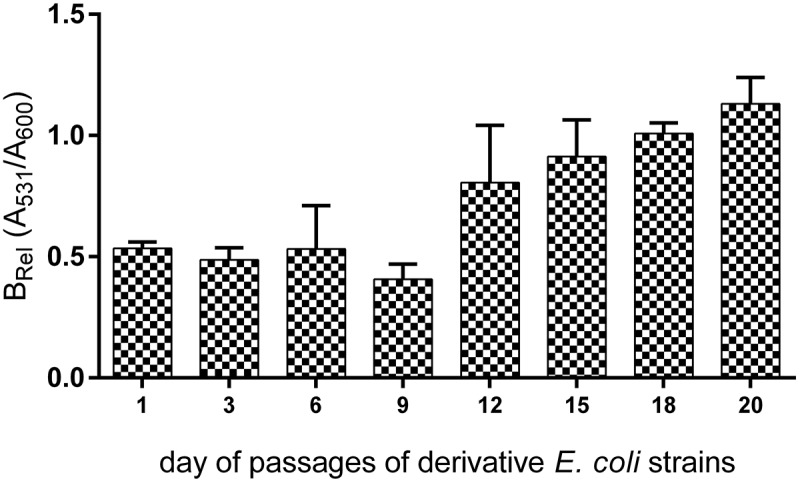
The stability of biofilm formation of the amoxicillin-induced derivatives of *E. coli* No. 5 and No. 6. The biofilm formation was measured after the subsequent days of culture passages under the control condition (without antibiotic). The biofilm was analyzed based on the OD value of the absorbed crystal violet (0.3%) measured at 531 nm (A_531_). The results represent the values of relative biofilm formation (B_rel_) independent of bacterial growth (A_531_/A_600_). The study was performed in four replications in two independent experiments.

The level of biofilm formation was checked for all generated derivative strains (121) in the optimal condition culture (without the antibiotic). The wild-type strains exhibited a very low (on average 0.085) level of absorbed crystal violet and it was close to the negative control of biofilm (0.04). The obtained results were compared between the derivatives and their wild types of *E. coli* strains. Only derivatives of *E. coli* No. 5 and No. 6 induced by amoxicillin revealed the increase of biofilm formation (Figure 5). The level of biofilm formation among derivative strains was not equal but statistically significantly higher (up to 4 times) than biofilm formation among the wild types of *E. coli* strains. The derivatives generated in the following days of passage increased the biofilm formation gradually (Figure 6) and it was independent from the bacterial growth level. Furthermore, the optical density of the growth (A_600_) was on the same level in both derivatives and wild-type strains. Additionally, the six representative derivative strains with increased biofilm formation (*E. coli* No.: 5/13AML, 5/17AML, 5/21AML, 6/9AML, 6/15AML, and 6/21AML) were selected for the analysis of the biofilm formation stability. The bacterial cultures were passed for 20 days in control conditions (without antibiotics). The derivative strains demonstrated a stable biofilm formation, which showed an additional increase from the 12^th^ day of passage (Figure 7).

### DNA fingerprints

**Figure 8.**
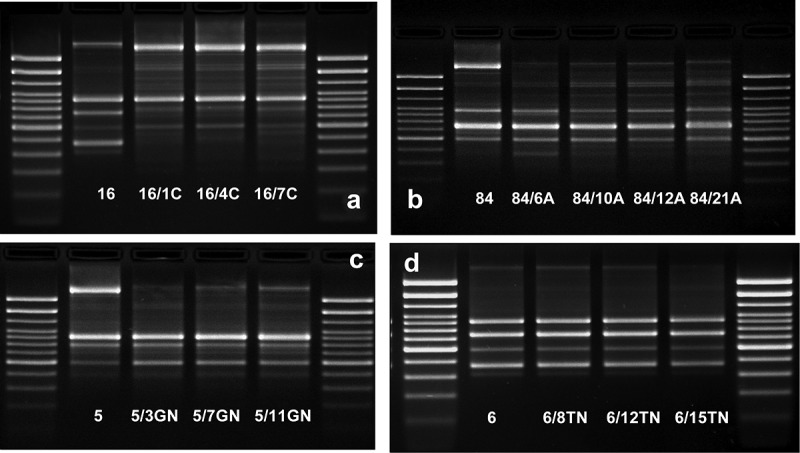
The comparison of the fingerprint profiles of the CGG-PCR products amplified from *E. coli* wild type (No. 1, No. 5, No. 6, No. 16, and No. 84) and their antibiotic-induced derivatives (exemplary pictures): C – ciprofloxacin-induced *E. coli* derivatives, A – amoxicillin-induced *E. coli* derivatives, GN – gentamycin-induced *E. coli* derivatives, TN – tobramycin-induced *E. coli* derivatives. The external DNA band patterns – 100 bp DNA Ladder (Invitrogen).

All 121 derivatives were compared to their wild-type *E. coli* strains based on the patterns of the CGG-PCR products. Figure 8 shows examples of the comparison of band patterns between the *E. coli* wild types and their derivatives generated by different antibiotics. Generally, the band patterns of aminoglycoside-induced derivatives were the most similar to their wild-type strains. The most visible differences were observed between the wild types and their ciprofloxacin-induced derivatives. The degree of differentiation of DNA fingerprints was directly proportional to other observed changes among *E. coli* derivatives. However, *E. coli* No. 84 was the most stable strain. The slight changes of sensitivity to antibiotics and similarly small changes of the CGG fingerprints appeared among its derivatives. The passage of *E. coli* wild-type cultures in optimal conditions was conducted for 4 days. The strains preserved the same band patterns of the CGG-PCR product as before the passes.

## Discussion

Currently, bacterial resistance to antimicrobials is widely explored, including not only typical mechanisms of drug resistance, but it is also considered in the context of complex bacterial pathogenicity [8]. Bacterial adaptation to the environment is one of the oldest mechanisms of living cells in the world, so it can be assumed that bacteria can regulate it via different pathways of metabolism. That is why we have put forward a hypothesis that antibiotics can also induce global changes in the uropathogenic *Escherichia coli* cells. Generally, we wanted to investigate what the consequences would appear during long-term exposure of the UPEC strains to sublethal concentrations of different antibiotics. Similar investigations have been conducted for a long time on many different bacterial species [8,34–37]. However, the effect of antibiotics on different metabolic pathways in bacteria still needs to be clarified. The bacterial exposure on the sub-MIC of antibiotics is an important factor for emergence of bacterial drug resistance, especially since it can happen during the antibiotic treatment of a bacterial infection or during preventive use of antibiotics in animal breeding [38,39].

### Antibiotics induce different antibiotic susceptibility changes

First, the generation rate of derivatives, the changes in the drug-resistance profiles and the stability of the acquired resistance were analyzed among selected *E. coli* derivatives. Almost all studied *E. coli* strains acquired the drug resistance during the treatment with the sub-MIC of all used antibiotics. Only one *E. coli* strain did not generate any derivatives resistant to ciprofloxacin and it was the most stable bacterial strain. Ciprofloxacin induced the resistance the fastest (just after the first day of passage) and resistance to all fluoroquinolones was observed in all derivative strains. The similar observation was described by Soto et al. [34]. This resistance is quickly generated by mutation in *gyrA* and *parC* [40–42] that is irreversible and strongly correlated with all fluoroquinolones. This corresponds with the previously presented synergistic effect of antibiotics detected with Cohen’s kappa correlation, where the resistance to one fluoroquinolone was compatible with the resistance to all fluoroquinolones [33]. Additionally, the observed correlation between simultaneous acquisition of the ciprofloxacin-induced resistance to fluoroquinolones, amoxicillin/clavulanate and trimethoprim indicates the dangerous mechanism of cross-resistance among the UPEC strains. That cross-resistance could be correlated with the induction of the efflux pump, which removed the antibiotics from the cell [43]. Chang et al. [44] suggested that the mutations in *gyrA* or *parC* were strongly correlated with the overexpression of the AcrAB efflux pump and resistance to betalactams, including clavulanic acid. Similar correlations of the increased expression of AcrAB/TolC and the decreased expression of OmpC in the ciprofloxacin-resistant mutant of *S. typhimurium* were presented by Fabrega et al. [45]. The role of the ArcAB-TolC in the induction of multidrug resistance was described also by other authors [46,47]. The exclusion of fluoroquinolones from routine treatment in outpatient therapy and searching for alternatives should be considered. The application of fluoroquinolones is becoming increasingly questionable and the limitation of their use is promoted by many scientists [7,9–12,48,49]. In turn, ciprofloxacin induced cross-resistance to the smallest number of antibiotics in comparison to others.

In the case of other antibiotics – amoxicillin also induced the resistance very quickly (just after the second day of passage) and it generated the most numerous groups of derivatives. However, amoxicillin induced the resistance to other betalactams only in approx. 25% of derivatives, but the resistance to amoxicillin/clavulanate appeared in 85% of this group of derivatives. Considering the stability of the induced resistance, the derivatives lost the resistance to amoxicillin or cefoxitin during the passage of culture in optimal conditions (without the amoxicillin), whereas the resistance to amoxicillin/clavulanate was irreversible, which was also observed in the case of ciprofloxacin-induced resistance. The induction of the overexpression of the chromosomal beta-lactamase AmpC in the case of *E. coli* is not possible because of the lack of the *ampR* gene, which is involved in the activation of *ampC* transcription. Therefore, AmpC in *E. coli* is noninducible but is controlled by the promoter and attenuator mechanisms [50]. These observations could probably be a result of ArcAB overexpression as it was mentioned above. Another possibly induced mechanism might be the return to the original membrane structure with the OmpC porins expression. However, the strong conclusion concerning the technical aspects seems to play an important role for this observation. During the analyses of the diameter zones of bacterial growth inhibition, they were similar around the amoxicillin disc and the amoxicillin/clavulanic disc, and the sensitivity to these antibiotics was strongly decreased in comparison to the wild type strains. Thus, these differences can arise from the technical properties of clinical breakpoint tables from EUCAST, where the norms of classification of bacteria in terms of resistance in the case of amoxicillin and amoxicillin/clavulanic are different. Finally, only the resistance to imipenem did not appear in any derivatives. All our previous and current studies proved that imipenem seems to be the most effective antibiotic against the UPEC strains. Similar observations were presented by other authors [51]. Imipenem belongs to the broad-spectrum pf antibiotics combating aerobic and anaerobic Gram-positive and Gram-negative pathogens. Its effectiveness also reinforces the resistance to betalactamases. Betalactam antibiotics are presented as safer than others for treatment and currently they are the gold standard for antibiotic therapy. However, the increasing emergence of carbapenem-resistant bacterial strains producing new variants of NDM betalactamase is well known [52–55]. In our study, besides the rapidly emerging antibiotic tolerance, amoxicillin induced cross resistance to the largest number of antibiotics. The routine and empirical application of betalactams is not a good solution, taking into account these findings and the constantly emerging new beta lactamases with an increasingly broad spectrum of their activity. The least influential antibiotics seem to belong to aminoglycosides, of which the rarest induce cross-resistance in the *E. coli* derivatives. These observations result from other drug-resistance mechanisms that are associated with translation and the ribosomal structure [56,57]. Additionally, we also observed the statistically significant increased susceptibility to ciprofloxacin in correlation to resistance to gentamycin. It may result from their antagonistic mechanisms of drug-resistance. Suzuki et al. [58] proved that resistance to aminoglycosides reduces proton-motive force that decreases the AcrB expression, leading to susceptibility to drugs which bacteria cannot exclude from the cell. Interestingly, we also observed an opposite phenomenon, wherein the induction of resistance to ciprofloxacin caused increased sensitivity to gentamycin and netilmicin. Maybe that reverse dependence can be connected with increased expression of the acrAB–TolC efflux system. Atac et al. [59] proved that among *E. coli* ST_131_ strains, the *marA* overexpression was correlated to the resistance to quinolones. What is more, the gentamicin resistance was statistically lower in ST_131_ than in non-ST_131_. In our research, amoxicillin also revealed similar dependence, but only in the case of netilmicin. Generally, it is known that fluoroquinolones, betalactams and aminoglycosides can act as synergists, so this intriguing observation can be a starting point for further studies. This is the first time the phenomenon of that antagonistic relationship between ciprofloxacin and gentamycin has been observed.

In conclusion, one antibiotic can lead to numerous cross-resistances emerging among *E. coli* strains. All four antibiotics induced the resistance to the other antibiotics from all six analyzed classes, except ciprofloxacin, which did not induce the resistance to aminoglycosides and imipenem. As we mentioned above – it can result from various mechanism of drug resistance that could be induced by the stress-response of the *E. coli* cell. Antibiotic-induced stress inside the bacterial cell can change the gene expression profile and it regulates the bacterial metabolism, it also has an impact on other virulence properties of bacteria [59,60].

### Ciprofloxacin induces the loss of the virulence factor genes to varying degrees

The subsequent important stage of our study was to verify the extent to which the antibiotics are able to influence the virulence genes of the UPEC strains. It has been long observed that antibiotic resistance correlates with certain bacterial features, this correlation is especially observed between fluoroquinolones and bacterial virulence [27,28,34,61–67]. In our study, the presence of six virulence factor gene regions (*papC, sfaE/D, cnf1, usp, fimG/H, hlyA*) and their expression were analyzed. These genes (except *fimG/H*) are described in the literature as specific for the UPEC strains [16–18,20]. It is interesting that the presence of specific virulence-associated genes and deep comprehensive phylogenetic analysis distinguishes UPEC from many commensals and intestinal pathogenic *E. coli* strains [19,68,69]. Most of these urovirulence genes are carried on the Pathogenic Islands (PAIs) [70–75]. One analyzed gene-fragment – *fimG/H*, encodes subunits of Fimbria Type I. That fimbria is very important at the early stage of UTI, but it is very commonly present for all type of *E. coli* strains and it is chromosomally encoded [76,77]. Their stable position in the *E. coli* genome seems to be important to ensure the primary adhesive property. This was probably the reason *fimG/H* were preserved in all derivative strains in our study and that fimbria is rather like a fitness, not a virulence factor [69].

In case of the urovirulence factor genes – only ciprofloxacin induced the loss of these genes. *E. coli* No. 87 did not acquire the resistance to ciprofloxacin, which was equivalent with conservation of all virulence factor genes. It is worth to add that it was the only one strain with all six analyzed virulence factor genes. This could mean that the presence of these genes was stabilized and the mobility properties of the PAIs were lost. In the event of other *E. coli* strains – the loss of virulence genes appeared just after the first day of passage, but we can see a clear difference between *E. coli* No. 6 and No. 16. Almost all derivatives of *E. coli* No. 6 lost all the analyzed genes and one of them saved all the genes, whereas derivatives of *E. coli* No. 16 have lost their genes differently. The loss of all genes together may indicate the presence of one pathogenic island, however, none of the known PAIs have all the studied genes (*sfaE/D, cnf1, usp*, and *hlyA*) at once. The observed simultaneous loss of *hlyA* and *cnf1* in all derivatives of *E. coli* No.6 can indicate the loss of the PAI II_J96_. Additionally, the loss of sfaE/D and *usp* can accordingly reflect the presence of PAI III_536_ and small usp-specific PAIs in *E. coli* No. 6 genome. In case of *E. coli* No. 16, the identification of specific PAIs with *pap* genes is difficult because of their potential presence on many PAIs (PAIs_J96_, PAIs_CFT073_), while the *sfa* are present on PAI III_536_ and *usp* represents specific small PAIs [78–81]. The tendency to maintain the genes in *E. coli* No. 16 can indicate the loss of some mobile genetic elements and increased stabilization of PAI regions in the genome in comparison to *E. coli* No. 6.

Although the correlation between the presence of the urovirulence genes and antimicrobial resistance susceptibility was often described, the effects of long-time pressure of sublethal antibiotics concentrations on the UPEC strains has not been clarified. Some sources indicate that DNA repair mechanisms are related to this phenomenon. A similar study was presented by Soto et al. [34], they also observed the simultaneous loss of *hly* and *cnf1* in all studied UPEC strains just after the first day of passage. In contrast to our results, they did not observe the loss of *pap* and *sfa* genes. Sanchez-Cespedes et al. [42] observed that *gyrA* mutation decreased the expression of *fimA, papA, papB,* and *ompA*. This mechanism is probably related to a change of DNA topology, which disrupted the normal gene expression process. The gyrase expression plays an important role here, which can relax the DNA helix and lead to mutational changes in bacterial genome via DNA-repair systems [82–84]. However, the observed phenomenon [36,42] indicate that SOS activation is not necessary for the loss of virulence factor genes induced by ciprofloxacin. Perhaps the observed duality of the results arises from other mechanisms of DNA repair like the Double-Strand break repair, mismatch repair, or antibiotic-induced competence for transformation in response to stress [8,85,86]. Maybe it is worth to consider that a different DNA sequence of mobile elements of different PAIs can be of significance to this study.

### Amoxicillin increases biofilm formation

The examination of the antibiotics’ influence on bacterial biofilm has been frequently described [37]. These observations concerned analysis in real time of bacterial incubation with antibiotic. We present for the first time how an antibiotic can permanently change the ability to form biofilm among the UPEC strains. Only amoxicillin-induced derivatives of *E. coli* strains demonstrated a statistically significant higher level of biofilm formation and what is important, it was not dependent on the density of planktonic cells. What should be emphasized – the wild types of the analyzed *E. coli* strains exhibited a very low level of absorbed crystal violet during the biofilm formation assay, whilst after the treatment with amoxicillin – their selected derivative strains demonstrated up to four times higher volume of absorbed crystal violet. This may indicate that biofilm formation can be induced by amoxicillin even in the strains unable to create biofilm. This situation can be very adverse during UTI treatment, where a sublethal concentration of amoxicillin in the urinary tract can lead not only to the selection of resistant cells but facilitate bacterial adhesion to the uroepithelial tissue of the host and help develop bacterial biofilm. Amoxicillin belongs to antibiotics that affect the bacterial cell wall structure and induce bacterial stress, which can stimulate biofilm formation [87]. The changes of bacterial cell surface can have an effect on their hydrophobicity and in consequence on biofilm formation [88,89]. Similar extensive studies have been carried out by Goneau et al. [36]. They described an *in vivo* study, in which the subinhibitory antibiotics (ciprofloxacin, ampicillin and gentamicin) modulated the virulence in the uropathogens, *inter alia* the *Escherichia coli*. The induction of the expression of adhesins caused an increase in biofilm formation, colonization of the murine bladder and the kidneys, and promoted intracellular bacterial community. A similar observation *in vitro* in other bacterial species has also been described in the literature. The study on *Staphylococcus saprophyticus* revealed that the sub-MIC of ciprofloxacin increased the bacterial adherence to glass microscope slides, ureteral stent material and bladder cell monolayers [90]. Kaplan et al. [91] observed 10-fold biofilm increase induced by subminimal inhibitory concentrations of β-lactam antibiotics added to a MSRA strains culture. They proved that it was dependent on cell lysis and in consequence the DNA released into the environment was utilized for the construction of matrix biofilm [92,93]. Taking into account our results – the growth curves of derivatives were not disturbed, so the increase of biofilm formation did not result from the increase of lysed bacterial cells. It could also result from the amoxicillin-induced resistance of the studied derivative strains. Mlynek et al. [93] suggest that amoxicillin stimulate extracellular DNA-dependent biofilm formation in bacteria, which can reflect an adaptation to cell wall stress. Another study [94,95] presents the increase of biofilm formation induced also by β-lactam antibiotics in the *Pseudomonas aeruginosa* culture. To sum up, this research shows the increase of biofilm during treatment with betalactams, while our results present a stabile increase of biofilm formation after the treatment with amoxicillin. Furthermore, the derivative strains continued the increase of biofilm formation in subsequent days of passage without amoxicillin, which can suggest the induction of some specific gene expressions and their following overexpressions.

### Antibiotics induce changes of CGG profiles

On the last stage of the study we genotyped the derivatives and their wild types of the studied *E. coli* strains via the CGG-PCR developed in our previous study [30]. This technique maps the genomic fingerprints of the studied strains. In the previous study, the designed CGG-PCR have divided the studied *E. coli* strains into two groups with different pathogenicity. On the other hand, the CGG-PCR indicated the subtle differences specific to individual strains. Following these achievements, we wanted to look into the genomes of *E. coli* derivatives using the same method. The primary band patterns of the CGG-PCR products were preserved in the *E. coli* derivatives compared to their wild type strains, but the differences were also observed by disclosure or disappearance of single bands. These differences corresponded the most with ciprofloxacin-induced derivatives, especially where the loss of virulence factor genes was also observed. However, slight differences were also observed after the treatment with amoxicillin. These findings are justifiable because of the strong influence of ciprofloxacin on DNA and its metabolism [34,36,82–85]. Amoxicillin has a weaker impact, but its ability to induce free radicals can affect DNA [25]. The stable band patterns of aminoglycoside-induced derivatives results from the lack of an aminoglycoside influence on the DNA structure [56,57]. It is worth to add that observed changes of the CGG-PCR band patterns were induced by antibiotics, because no changes were not observed after the passages of the culture in optimal conditions. The method of genotyping via MLEE or ribotyping of the rDNA via RFLP are standards used for bacterial differentiation, however they require extensive laboratory experience and they are unable to differentiate the bacterial pathogenicity [96]. These results confirm the previous conclusion that the CGG-PCR may be a useful technique for epidemiological investigation of kinship between *E. coli* strains.

To summarize, our study provides a broad description of the correlation between sublethal antibiotic treatment and cross-resistance acquisition, virulence factor gene loss, and the increase of biofilm formation among UPEC strains. Similar observations in other bacteria species were also presented by different authors. Dewan et al. [97] revealed that *Bordetella bronchiseptica* rapidly developed stable and persistent macrolide resistance but it lost virulence and the ability to colonize mice. Likewise, the treatment of the *Acinetobacter baumannii* culture with subinhibitory concentrations of imipenem increased biofilm formation, motility, and type IV pili synthesis [98,99], compared to a treatment with carbapenems that decreased the expression of virulent *omp* [100]. The greatest and the most stable changes were observed in the case of ciprofloxacin, which confirms that ciprofloxacin has a strong influence on DNA metabolism and/or activates other pathways which bacteria use for adaptation in unfavorable environment. The bactericidal effect lasts the longest in the case of aminoglycosides. They induce the least changes in bacteria, which exhibit the highest sensitivity to them. Unfortunately, aminoglycosides cause the most numerous and the strongest side effects in humans in comparison to fluoroquinolones and betalactams. Our study exhibits the multiplex effect of antibiotics on the UPEC strains. This group of pathogens exhibit a high capacity to resistance to modern therapies. There are often responsible for frequent recurrent infections as well as fast drug-resistance build-up [49]. The ability to form an intracellular biofilm is a way to cause permanent presence in the urinary tracts of infected patients, which can lead to severe damage or destruction of this system. The understanding of antibiotic resistance mechanisms of the UPEC strains is a chance to develop better therapies. This can lead to both health-related and economic benefits and the presented results are an important signal to reflect on the restrictive use of antibiotics.

## Conclusions

The broad results revealed a few important observations related to UPEC strains. Firstly, drug resistance emerges immediately when antibiotic concentration decreases below the MIC, what can induce many cross-resistances to antibiotics from different classes. Additionally, the antagonistic effects between antibiotics were observed. It should be emphasized that the most noticeable changes among the studied bacteria were observed in case of amoxicillin and ciprofloxacin. Amoxicillin decreased the sensitivity to other betalactams and increased the sensitivity to netilmicin. Similarly, ciprofloxacin decreased the sensitivity to betalactams, but also to trimethoprim, trimethoprim-sulfamethoxazole. Furthermore, a strong antagonistic effect was observed between ciprofloxacin and aminoglycosides. It is worth to add that amoxicillin had no influence on the virulence genes but increased biofilm formation, contrary to ciprofloxacin – which induced the loss of virulence factor genes, but it had no influence on biofilm formation.
